# Effectiveness of the 2023 Autumn XBB.1.5 COVID‐19 Booster During Summer 2024 in the EU/EEA: A VEBIS Electronic Health Record Network Study

**DOI:** 10.1111/irv.70198

**Published:** 2025-11-28

**Authors:** James Humphreys, Nathalie Nicolay, Toon Braeye, Izaak Van Evercooren, Christian Holm Hansen, Ida Rask Moustsen‐Helms, Chiara Sacco, Massimo Fabiani, Jesús Castilla, Iván Martínez‐Baz, Ausenda Machado, Patricia Soares, Rickard Ljung, Nicklas Pihlström, Esther Kissling, Anthony Nardone, Susana Monge, Sabrina Bacci, Baltazar Nunes, VEBIS‐EHR working group, Joris Van Loenhout, Alberto Mateo‐Urdiales, Andre Brito

**Affiliations:** ^1^ Epiconcept Paris France; ^2^ Vaccine Preventable Diseases and Immunisation European Centre for Disease Prevention and Control (ECDC) Solna Sweden; ^3^ Sciensano Elsene Belgium; ^4^ Department of Infectious Disease Epidemiology and Prevention Statens Serum Institut Copenhagen Denmark; ^5^ Infectious Diseases Department Istituto Superiore di Sanità Rome Italy; ^6^ European Programme on Intervention Epidemiology Training (EPIET) European Centre for Disease Prevention and Control Stockholm Sweden; ^7^ Instituto de Salud Pública de Navarra—IdiSNA Pamplona Spain; ^8^ CIBER on Epidemiology and Public Health Madrid Spain; ^9^ Departamento de Epidemiologia e Centro de Estudos de Vectores e Doenças Infecciosas Dr. Francisco Cambournac Instituto Nacional de Saúde Doutor Ricardo Jorge Lisbon Portugal; ^10^ Swedish Medical Products Agency Uppsala Sweden; ^11^ Institute of Environmental Medicine Karolinska Institutet Stockholm Sweden; ^12^ Department of Communicable Diseases, National Centre of Epidemiology Institute of Health Carlos III Madrid Spain; ^13^ CIBER on Infectious Diseases Madrid Spain

**Keywords:** COVID‐19, electronic health records, vaccine effectiveness, XBB.1.5

## Abstract

**Background:**

After a period of low SARS‐CoV‐2 activity, viral circulation increased in Europe from May 2024, driven by immune‐evasive KP sublineages of the JN.1 variant. We estimated vaccine effectiveness (VE) of the XBB.1.5 dose administered in autumn 2023 against COVID‐19‐related hospitalisations and deaths in individuals 65 years of age or older during this period.

**Methods:**

We conducted a multi‐country cohort study across six EU nations in the VEBIS‐EHR network using linked electronic health records. VE against COVID‐19‐related hospitalisation and death during June–August 2024 was estimated using Cox regression in a two‐stage analysis, adjusting for demographics, comorbidities and prior vaccination history.

**Results:**

Among individuals 65–79 and ≥ 80 years old, respectively, VE of the XBB.1.5 dose ≥ 6 months post administration was 13% (95% CI: −12% to 33%) and 7% (95% CI: −7% to 19%) against hospitalisation and 39% (95% CI: −7% to 65%) and 3% (95% CI: −23% to 23%) against deaths.

**Conclusions:**

XBB.1.5 vaccination provided minimal residual protection against severe COVID‐19 outcomes among adults aged ≥ 65 years more than 6 months after vaccination, during the summer 2024 period of increased SARS‐CoV‐2 activity.

## 1 Introduction

After the official end of the COVID‐19 pandemic, SARS‐CoV‐2 continues to circulate year‐round due to shifting predominance of lineages and sublineages [1]. In Europe, following a period of very low activity after the winter of 2023–2024, SARS‐CoV‐2 test positivity began to increase in May 2024. Between May and June 2024, the KP sublineages of JN.1 became predominant in the European Union (EU) [2, 3]. Although the SARS‐CoV‐2 KP.2 and KP.3 sublineages were not expected to increase severity [2], concerns focused on their potential for immune escape compared with previously circulating XBB.1.5‐like lineages [4, 5, 6].

In most EU countries, there was no recommendation for an additional COVID‐19 dose in spring 2024. Given waning vaccine effectiveness (VE) and the potential for immune escape, the mismatch in the vaccine component received during the 2023 autumn campaigns and sublineages circulating in spring (BA.2.86/JN.1), protection conferred by the monovalent XBB.1.5 dose was expected to be low in the subsequent months [5, 6, 7].

A surge in hospitalizations, ICU admissions and deaths related to laboratory‐confirmed COVID‐19 followed, with large proportions of these severe cases occurring among individuals aged 65 years and older [1]. The exact timing of the peak in COVID‐19 deaths and admissions spanning summer 2024 varied by country, though it generally began about 8–9 months after the start of the 2023 autumn vaccination campaigns, during which the monovalent XBB.1.5 dose was most frequently administered [8].

Our objective was to estimate VE of the autumn XBB.1.5 dose against COVID‐19‐related hospitalisations and deaths occurring between June and August 2024, among individuals eligible for vaccination, aged 65 years or older and residing in six EU countries.

## 2 Methods

This VE study was undertaken within the VEBIS‐EHR network (Vaccine Effectiveness Burden and Impact Studies using Electronic Health Records), a multi‐country study funded by the European Centre of Disease Prevention and Control (ECDC) with six participating countries in the current analysis: Belgium, Denmark, Italy, Spain (Navarre), Portugal and Sweden.

Detailed methods are available from the study protocol for the 2023–2024 season and previous publications [7, 9, 10]. Briefly, using a common protocol, historical cohorts were constructed by each site, using deterministic linkage of electronic health records (EHRs). We included individuals aged 65 years or older and eligible to receive the 2023 autumnal COVID‐19 dose according to site recommendations. VE was estimated for the period between 1 June and 25 August 2024 using data extracted in October 2024 [9, 11].

Individuals eligible for vaccination (Supporting Information, Definition 1) contributed to unvaccinated person‐time at risk if they had not been vaccinated with the 2023 autumn booster between the start and end of the vaccine campaign, as defined locally. Vaccinated individuals contributed to vaccinated person‐time at risk after 14 days post vaccination had elapsed. Individuals' person‐time at risk stopped at the date of the outcome, the date of death for any cause, on the date of a subsequent vaccine dose during the autumn campaign or at the end of the study period (25 August 2024).

Individuals eligible for vaccination (Supporting Information, Definition 1) contributed to unvaccinated person‐time at risk if they had not been vaccinated with the 2023 autumn booster between the start and end of the vaccine campaign, as defined locally. Vaccinated individuals contributed to vaccinated person‐time at risk after 14 days post vaccination had elapsed. Individuals' person‐time at risk stopped at the date of the outcome, the date of death for any cause, on the date of a subsequent vaccine dose during the autumn campaign or at the end of the study period (25 August 2024).

Hospitalisation due to COVID‐19 was defined as a hospital admission due to a severe acute respiratory infection with a SARS‐CoV‐2 positive test from 14 days before to 1 day after admission or with COVID‐19 as the main diagnosis in hospital admission or discharge records. A COVID‐19‐related death was defined as death with the main cause coded as COVID‐19 and/or with a SARS‐CoV‐2 positive laboratory result in the 30 days preceding death.

Each study site used proportional hazards Cox regression models, with vaccination status as a time‐varying exposure and calendar time as the underlying scale, to estimate vaccine hazard ratios (aHR) adjusted by 5‐year age group, sex, comorbidities and previous number of vaccine booster doses, with other covariates included according to study site‐specific protocols. We pooled study site‐specific hazard ratios using a random‐effects meta‐analysis. We estimated pooled VE as one minus the pooled hazard ratio estimates expressed as a percentage (VE = (1 − pooled aHR) * 100), with heterogeneity scored using the *I* ^2^ index. Analysis was stratified by age group (65–79 years old and 80 and older).

## 3 Results

We included 19.3 million participants in this analysis. At the end of the study period, 13.3 million participants were unvaccinated and 6.0 million participants had received the XBB.1.5 vaccine during autumn 2023. Among those vaccinated, 99.8% of the participants had received the vaccine more than 6 months earlier (Table 1). Compared with unvaccinated participants, those who had received the monovalent XBB.1.5 vaccine presented a higher prevalence of high‐risk comorbidities (6.6% vs. 2.1%) and a higher proportion of those with at least two booster doses before the start of the autumn 2023 campaign (92.7% vs. 32.5%).

**TABLE 1:** Descriptive characteristics of the study population (*N* = 19,306,009) by vaccination status and time since vaccination at the end of the study period,^a^ within the six study sites (Belgium, Denmark, Italy, Navarre‐Spain, Portugal and Sweden), from 1 June 2024 to 25 August 2024, VEBIS‐EHR network.

Variable	Not vaccinated	Vaccinated ≥ 14 days	Vaccinated 14–89 days	Vaccinated 90–179 days	Vaccinated ≥ 180 days
	*n* (%)	*n* (%)	*n* (%)	*n* (%)	*n* (%)
Total (row % over total = 19,306,009)	13,264,417 (68.7)	6,041,592 (31.3)	67 (0.0)	13,455 (0.1)	6,028,070 (31.2)
Study site					
Belgium	1,009,248 (7.6)	1,091,923 (18.1)	0	0 (0.0)	1,091,923 (18.1)
Denmark	211,557 (1.6)	878,421 (14.5)	0	77 (0.6)	878,344 (14.6)
Italy	10,580,504 (79.8)	1,279,075 (21.2)	0	4649 (34.6)	1,274,426 (21.1)
Navarre	44,974 (0.3)	81,247 (1.3)	0	655 (4.9)	80,592 (1.3)
Portugal	936,703 (7.1)	1,269,745 (21.0)	67 (100)	4981 (37)	1,264,697 (21.0)
Sweden	481,431 (3.6)	1,441,181 (23.9)	0	3093 (23)	1,438,088 (23.9)
Age group (years)					
65–79	9,434,356 (71.1)	4,130,612 (68.4)	44 (65.7)	8951 (66.5)	4,121,617 (68.4)
≥ 80	3,830,061 (28.9)	1,910,980 (31.6)	23 (34.3)	4504 (33.5)	1,906,453 (31.6)
Sex					
Male	7,417,948 (55.9)	3,203,628 (53.0)	37 (55.2)	7049 (52.4)	3,196,542 (53.0)
Female	5,846,443 (44.1)	2,837,362 (47.0)	30 (44.8)	5829 (43.3)	2,831,503 (47.0)
Missing	26 (0.0)	602 (0.0)	0 (0.0)	577 (4.3)	25 (0.0)
Comorbidities^b^					
High risks	280,976 (2.1)	400,625 (6.6)	18 (26.9)	1292 (9.6)	399,315 (6.6)
Medium/low risk	4,702,692 (35.5)	3,058,940 (50.6)	30 (44.8)	6317 (46.9)	3,052,593 (50.6)
No	8,251,984 (62.2)	2,565,887 (42.5)	15 (22.4)	5266 (39.1)	2,560,606 (42.5)
Missing	28,765 (0.2)	16,140 (0.3)	< 5 (6.0)	580 (4.3)	15,556 (0.3)
Number of previous booster doses					
0	1,403,530 (10.6)	29,279 (0.5)	0 (0.0)	196 (1.5)	29,083 (0.5)
1	7,546,294 (56.9)	411,648 (6.8)	10 (14.9)	1724 (12.8)	409,914 (6.8)
2	3,898,637 (29.4)	3,831,236 (63.4)	41 (61.2)	7489 (55.7)	3,823,706 (63.4)
3	397,923 (3.0)	1,420,043 (23.5)	11 (16.4)	3107 (23.1)	1,416,925 (23.5)
4	17,983 (0.1)	347,173 (5.7)	0 (0.0)	358 (2.7)	346,815 (5.8)
5	46 (0.0)	1599 (0.0)	0 (0.0)	0 (0.0)	1599 (0.0)
Missing	< 5 (0.0)	614 (0.0)	5 (7.4)	581 (4.3)	28 (0.0)
Vaccine product					
Pfizer (XBB.1.5)	—	5,942,540 (98.4)	67 (100%)	12,650 (94.0)	5,929,823 (98.4)
Moderna (XBB.1.5)	—	49,089 (0.8)	0 (0.0)	0 (0.0)	49,089 (0.8)
Novavax (XBB.1.5)	—	5165 (0.1)	0 (0.0)	220 (1.6)	4945 (0.1)
Others	—	44,177 (0.7)	0 (0.0)	0 (0.0)	44,177 (0.7)
Missing	—	621 (0.0)	0 (0.0)	585 (4.3)	36 (0.0)

^a^ Vaccination status and time since vaccination were assessed at the end of the individual observation period.

^b^ High‐risk comorbidities: immunocompromised conditions with COVID‐19 vaccine recommendation; medium/low risk: nonimmunocompromised conditions with COVID‐19 vaccine recommendation; no comorbidities: persons without any of the risk comorbidities. Details are presented in Table S3.

Among participants aged 65–79 years, the unvaccinated and vaccinated groups had COVID‐19 hospitalisation rates of 6.2 per 100,000 person‐months (1664 hospitalisations over 26.7 million person‐months) and 6.4 per 100,000 person‐months (748/11.7 million person‐months), respectively. Corresponding COVID‐19‐related death rates were 0.9 per 100,000 person‐months (224/24.4 million person‐months) and 2.1 per 100,000 person‐months (191/9.3 million person‐months), respectively. Overall, XBB.1.5 VE against COVID‐19 hospitalisations was 13% (95% CI: −12% to 33%) (Figure S1). Stratified by time since vaccination, VE was 16% (95% CI: −52% to 54%) among those 90–179 days post vaccination and 13% (95% CI: −12% to 33%) among those ≥ 180 days post vaccination. Due to the low number of events, VE could not be estimated for the 14‐ to 89‐day interval. Against COVID‐19‐related deaths, VE was 38% (95% CI: −12% to 65%) for participants vaccinated ≥ 180 days ago (Figure S3). No other VE estimates could be produced by time since vaccination for the 65‐ to 79‐year age group (Table 2).

**TABLE 2:** Number of COVID‐19 hospitalisations and COVID‐19‐related deaths, person‐months at risk by vaccine status and VE overall and by time since vaccination for individuals aged 65–79 and ≥ 80 years old, within the six study sites (Belgium, Denmark, Italy, Navarre‐Spain, Portugal and Sweden), from 1 June 2024 to 25 August 2024, VEBIS‐EHR network.

	Events (person‐months)	Rate per 100,000 person‐months	VE (95% CI)	Heterogeneity *I* ^2^ (min–max study level VE estimates)
Age group 65–79				
Hospitalisations				
Not yet vaccinated	1664/26,728,241	6.2	ref	ref
Overall vaccinated (≥ 14 days)	748/11,669,302	6.4	13.2% (−11.8 to 32.6)	68.8% (−127%, BE to 39%, SE)
Vaccinated (14–89 days)	—	—	*NE* ^a^	—
Vaccinated (90–179 days)	12/329,669	3.6	16.2% (−51.5 to 53.6)	0% (−12%, PT to 36%, IT)
Vaccinated (≥ 180 days)	734/11,256,259	6.5	13.2% (−11.9 to 32.6)	68% (−127%, BE to 39%, SE)
Deaths				
Not yet vaccinated	224/24,375,169	0.9	ref	
Overall vaccinated (≥ 14 days)	191/9,293,458	2.1	38.5% (−7.4 to 64.8)	64.5% (12%, PT to 77%, SE)
Vaccinated (14–89 days)	—	—	*NE* ^a^	—
Vaccinated (90–179 days)	—	—	*NE* ^a^	—
Vaccinated (≥ 180 days)	187/8,888,869	2.1	37.5% (−12.1 to 65.2)	67.1% (10%, PT to 77%, SE)
Age group ≥ 80 years				
Hospitalisations				
Not yet vaccinated	2329/10,645,955	21.9	ref	ref
Overall vaccinated (≥ 14 days)	1124/4,522,993	24.9	7.1% (−6.8 to 19.2)	31.9% (−22.1%, NV to 24%, SE)
Vaccinated (14–89 days)	—	—	*NE* ^a^	—
Vaccinated (90–179 days)	28/195,612	14.3	12.5% (−28.8 to 40.5)	0% (−1%, SE to 19%, IT)
Vaccinated (≥ 180 days)	1095/4,320,527	25.3	7.2% (−7.2 to 19.7)	34.5% (−21.2%, NV to 25%, SE)
Deaths				
Not yet vaccinated	563/10,089,020	5.6	ref	ref
Overall vaccinated (≥ 14 days)	585/3,595,232	16.3	3.4% (−21.4 to 23.2)	33.1% (−21%, IT to 37.8%, DK)
Vaccinated (14–89 days)	—	—	*NE* ^a^	—
Vaccinated (90–179 days)	9/30,829	29.2	37.0% (−23 to 67.7)	0% (37%, PT)
Vaccinated (≥ 180 days)	574/3,394,933	16.9	2.6% (−23.4 to 23.2)	35.7% (−23%, IT to 37.6%, DK)

*Note:* VE = one minus the pooled confounder‐adjusted hazard ratio * 100% at study level using a Cox regression time dependent model (confounder variables used in each study site are available in Annex 3 and 4 of the Supporting Information).

Abbreviations: BE: Belgium, CI: confidence interval, DK: Denmark, IT: Italy, NV: Navarre, Spain, PT: Portugal, SE: Sweden, VE: vaccine effectiveness.

^a^ NE: not estimable due to insufficient events (< 15).

Among participants aged ≥ 80 years, COVID‐19 hospitalisation rates were 21.9 per 100,000 person‐months (2329/10.6 million person‐months) in the unvaccinated group and 24.9 per 100,000 person‐months (1124/4.5 million person‐months) in the vaccinated group. COVID‐19‐related death rates were 5.6 per 100,000 person‐months (563/10.1 million person‐months) among the unvaccinated and 16.3 per 100,000 person‐months (585/3.6 million person‐months) in the vaccinated group (Table 2). For those ≥ 180 days post vaccination, VE was 7% (95% CI: −7% to 19%) against hospitalisations and 3% (95% CI: −21% to 23%) against deaths (Figures S2 and S4). For those vaccinated 90–179 days earlier, VE was 13% (95% CI: −29% to 41%) against hospitalisations and 37% (95% CI: −23% to 68%) against deaths.

Heterogeneity between estimates produced at the study‐site level and later pooled via random effects was moderate (*I* ^2^: 65%–69%) for the 65‐ to 79‐year age group and low (*I* ^2^: 0%–36%) for those ≥ 80 years (Table 2).

## 4 Discussion

Within the VEBIS‐EHR network, we estimated null or very low XBB.1.5 VE among individuals aged ≥ 65 years between June and August 2024. Nearly all (99.8%) vaccinated participants had received their dose at least 6 months prior. During the study period, XBB.1.5 VE against COVID‐19 hospitalisations was 13% (95% CI: −12% to 33%) among those aged 65–79 years and 7% (95% CI: −7% to 19%) among those ≥ 80 years. Against COVID‐19‐related deaths, VE was 39% (95% CI: −7% to 65%) in the 65‐ to 79‐year age group and 3% (95% CI: −21% to 23%) in those aged ≥ 80 years.

These findings are consistent with two studies examining XBB.1.5 VE against COVID‐19 hospitalisation during summer 2024 in older populations [12, 13]. In a Canadian study covering 10 months following the start of the 2023 vaccination campaign, XBB.1.5 vaccination conferred null or very low (< 14%) protection ≥ 7 months post vaccination, including during spring/summer 2024 [12]. Similarly, the UK Health Security Agency (UKHSA) reported autumn 2023 VE estimates of 32% (95% CI: 11%–48%), 25% (95% CI: 15%–35%) and 12% (95% CI: 4%–20%) for individuals who were 20–24 (140–168 days), 25–29 (175–203 days) and ≥ 30 weeks (210 days) post vaccination, respectively, in April 2024 [13].

The lower XBB.1.5 VE observed among older EU residents during summer 2024 may be due to waning protection beyond 6 months [7, 12], as well as antigenic mismatches between the XBB.1.5 vaccine component and the Omicron BA.2.86 sublineages (including JN.1) circulating between June and August 2024 [5, 6] (Supporting Information). In two previous VEBIS‐EHR studies that estimated VE during XBB.1.5 [9] and BA.2.86/JN.1 [10] predominance, we noted decreases in VE against severe outcomes, possibly reflecting both viral evolution and the natural waning of vaccine‐induced immunity.

Nonetheless, the XBB.1.5 vaccine continued to provide moderate protection during the BA.2.86 predominance period [10]. In England, where XBB.1.5 was offered as a 2024 spring booster dose, protection against hospitalisations in the first 2 months following vaccination returned to levels observed early in the 2023 autumn campaign, which supports waning immunity (rather than immune escape by emerging KP sublineages) as the primary explanation for declining protection [13].

Those that received the autumn XBB.1.5 dose had a greater number of previous boosters (92.7% with at least two boosters) compared with those that did not receive vaccination this season (32.5%). In addition, the prevalence of high‐risk comorbidities in those vaccinated (6.6%) was higher than in the unvaccinated cohort (2.1%). These figures suggest that there are growing differences in the characteristics of those who continue to vaccinate versus those who do not.

When interpreting these results, it is important to consider the limitations inherent to using secondary EHR data for VE research—namely, misclassification of vaccination status and outcomes and the absence of sufficient covariates for full confounder adjustment. Additionally, because we estimated VE approximately 8 months after the 2023 autumn campaign, there is a higher risk of bias from differential depletion of susceptibles by vaccination status [14, 15], possibly underestimating VE. This bias could partly explain the unexpected observation of higher COVID‐19 hospitalisation and death rates among vaccinated individuals (Table 2). However, simulation studies suggest that this bias alone is unlikely to fully account for the decline in VE with time since vaccination or the elevated incidence among vaccinated persons [16]. Further, estimates based on a small number of events, such as those against death among those aged 65–79, should be interpreted with caution.

There is an additional limitation impacting estimates in one participating country, Belgium, for which the national demographic dataset used to censor deaths in linked registry analyses has not been updated since July 2024. Records after this date may include some individuals who have since died as they can no longer be censored appropriately, potentially leading to an underestimation of VE against hospitalization. However, Belgium contributed a relatively small proportion of the total events across all participating sites in this analysis and is not included in estimates against COVID‐19 related mortality. Therefore, while this issue must be taken into account when interpreting the results, its overall impact on the multi‐country VE estimates is likely to be limited.

In conclusion, our multi‐country study indicates that beyond 6 months after administration, the XBB.1.5 vaccine provides minimal residual protection against severe COVID‐19 outcomes among older adults during the summer 2024 peak in COVID‐19 activity. These findings highlight the need for improved COVID‐19 vaccines offering longer‐lasting effectiveness. Should COVID‐19 circulation follow a predictable seasonal pattern, the results may support considerations for a spring booster dose in higher risk groups.

## Author Contributions

**James Humphreys:** conceptualization, investigation, writing – original draft, methodology, validation, visualization, writing – review and editing, software, formal analysis, project administration, data curation, supervision. **Nathalie Nicolay:** resources, funding acquisition, conceptualization, validation, writing – review and editing, project administration, supervision, methodology, investigation. **Toon Braeye:** conceptualization, investigation, writing – review and editing, methodology, validation, writing – original draft, data curation, formal analysis, supervision, funding acquisition. **Izaak Van Evercooren:** data curation, supervision, formal analysis, methodology, validation, writing – review and editing, conceptualization, investigation, writing – original draft, software. **Christian Holm Hansen:** conceptualization, investigation, funding acquisition, writing – original draft, methodology, validation, writing – review and editing, formal analysis, data curation, supervision, project administration. **Ida Rask Moustsen‐Helms:** data curation, software, formal analysis, methodology, validation, writing – review and editing, conceptualization, investigation, writing – original draft. **Chiara Sacco:** data curation, software, formal analysis, methodology, validation, writing – review and editing, conceptualization, investigation, writing – original draft. **Massimo Fabiani:** conceptualization, investigation, funding acquisition, writing – original draft, methodology, validation, writing – review and editing, data curation, supervision, resources, project administration, formal analysis. **Jesús Castilla:** conceptualization, investigation, writing – original draft, methodology, validation, software, data curation, supervision, resources, project administration, formal analysis, writing – review and editing, funding acquisition. **Iván Martínez‐Baz:** conceptualization, investigation, writing – original draft, methodology, validation, writing – review and editing, software, formal analysis, data curation, funding acquisition, supervision, resources, project administration. **Ausenda Machado:** conceptualization, investigation, writing – original draft, methodology, validation, writing – review and editing, software, formal analysis, project administration, data curation, supervision, resources, funding acquisition. **Patricia Soares:** conceptualization, investigation, writing – original draft, methodology, validation, writing – review and editing, software, formal analysis, data curation, project administration. **Rickard Ljung:** conceptualization, investigation, writing – original draft, funding acquisition, resources, data curation, supervision, software, formal analysis, methodology, validation, writing – review and editing. **Nicklas Pihlström:** conceptualization, investigation, writing – original draft, methodology, validation, writing – review and editing, software, formal analysis, data curation. **Esther Kissling:** conceptualization, investigation, funding acquisition, writing – original draft, methodology, validation, writing – review and editing, project administration, supervision, resources. **Anthony Nardone:** supervision, resources, project administration, methodology, validation, writing – review and editing, conceptualization, investigation, funding acquisition, writing – original draft. **Susana Monge:** conceptualization, investigation, funding acquisition, writing – original draft, data curation, supervision, resources, software, formal analysis, project administration, methodology, validation, visualization, writing – review and editing. **Sabrina Bacci:** conceptualization, investigation, funding acquisition, methodology, validation, writing – review and editing, project administration, resources, supervision. **Baltazar Nunes:** data curation, supervision, resources, software, formal analysis, project administration, methodology, validation, visualization, writing – review and editing, conceptualization, investigation, funding acquisition, writing – original draft. **VEBIS‐EHR working group:** conceptualization, investigation, funding acquisition, writing – original draft, methodology, validation, visualization, writing – review and editing, software, formal analysis, project administration, data curation, supervision, resources.

## Funding

All the public health organisations involved received funding from the European Centre for Disease Prevention and Control (ECDC) Implementing Framework Contract ECDC/2021/018 ‘Vaccine effectiveness and impact of COVID‐19 vaccines through routinely collected exposure and outcome using health registries’ (RS/2022/DTS/24104). In Portugal, this work was also supported by FCT—Fundação para a Ciência e Tecnologia, I.P. by project reference CEECINST/00049/2021/CP2817/CT0001 and DOI identifier https://doi.org/10.54499/CEECINST/00049/2021/CP2817/CT0001.

## Ethics Statement

All study sites participating in this study conformed with their respective national and EU ethical and data protection requirements. Ethical statements for each of the participating study sites:

- *Belgium*: Data linkage and collection within the data warehouse have been approved by the information security committee. The study was conducted in accordance with the Declaration of Helsinki. Ethical approval was granted for the gathering of data from hospitalised patients by the Committee for Medical Ethics from the Ghent University Hospital (reference number bc‐07507) and authorisation for possible individual data linkage using the national register number from the Information Security Committee (ISC) Social Security and Health (reference number IVC/KSZG/20/384). Linkage of hospitalised patient data to vaccination and testing within the LINK‐VACC project was approved by the Medical Ethics Committee UZ Brussels–VUB on 3 February 2021 (reference number 2020/523) and authorisation from the ISC Social Security and Health (reference number IVC/KSZG/21/034).
- *Denmark*: Only administrative register data were used for the study. According to Danish law, ethics approval is exempt for such research, and the Danish Data Protection Agency, which is dedicated ethics and legal oversight body, thus waives ethical approval for the study of administrative register data when no individual contact of participants is necessary, and only aggregate results are included as findings. The study is, therefore, fully compliant with all legal and ethical requirements, and there are no further processes available regarding such studies.
- *Navarre (Spain)*: The study was approved by Navarre's Ethical Committee for Clinical Research, which waived the requirement of obtaining informed consent.
- *Portugal*: The study received approval from the Ethical Committee and the Data Protection Officer of the Instituto Nacional de Saúde Doutor Ricardo Jorge. Given that data were irreversibly anonymised, the need for the participants' informed consent was waived by the Ethical Committee.
- *Italy*: This study, based on routinely collected data, will not be submitted for approval to an ethical committee because the dissemination of COVID‐19 surveillance data was authorised by the Italian law N. 52 of 19 May 2022, following the law decree N. 24 of 24 March 2022 (Article n. 13). Based on the same acts, the information on COVID‐19 vaccination was retrieved by the Italian National Institute of Health using data from the National Immunisation Information System of the Italian Ministry of Health. Because of the retrospective design and the large size of the population under study, in accordance with Authorisation n. 9 released by the Italian data protection authority on 15 December 2016, the individual informed consent was not requested for the conduction of this study.
- *Sweden*: The Swedish study is approved by the Swedish Ethical Review Authority (2020‐06859, 2021‐02186) and has conformed to the principles embodied in the Declaration of Helsinki. Consent to participate is not applicable as this is a register‐based study.

## Conflicts of Interest

The authors declare no conflicts of interest.

## Supporting information


**Figure S1:** Forest plot of autumn vaccination VE against hospitalisation related to COVID‐19 among those aged 65–79 years.
**Figure S2:** Forest plot of autumn vaccination VE against hospitalisation related to COVID‐19 among those aged 80 plus.
**Figure S3:** Forest plot of autumn vaccination VE against death due to COVID‐19 among those aged 65–79 years.
**Figure S4:** Forest plot of autumn vaccination VE against death due to COVID‐19 among those aged 80 plus.

## Data Availability

Authors cannot share the data used for this study, which should be requested from the data owner institutions following their respective procedures.
