# Supplementary material for: Effectiveness of the 2023 Autumn XBB.1.5 COVID‐19 Booster During Summer 2024 in the EU/EEA: A VEBIS Electronic Health Record Network Study
Source: Influenza Other Respir Viruses. 2025 Nov 28;19(12):e70198. doi: 10.1111/irv.70198 (PMC12981528; doi:10.1111/irv.70198)
Supplement: Supplementary file 1 — Figure S1: Forest plot of autumn vaccination VE against hospitalisation related to COVID‐19 among those aged 65–79 years. Figure S2: Forest plot of autumn vaccination VE against hospitalisation related to COVID‐19 among those aged 80 plus. Figure S3: Forest plot of autumn vaccination VE against death due to COVID‐19 among those aged 65–79 years. Figure S4: Forest plot of autumn vaccination VE against death due to COVID‐19 among those aged 80 plus. [file IRV-19-e70198-s001.docx]

**Supplementary material**

**Definition 1: Eligibility criteria**

The study population includes community-dwelling individuals ≥65 years of age in national EHR databases. The study population should be eligible for seasonal COVID-19 vaccination, including belonging to an age group for whom seasonal COVID-19 vaccination has been recommended in each site/country. Eligibility will be based on the following criteria as of the first day of the vaccination campaign, which may be adapted to match national recommendations:

● Aged between 65 and 110 years at the beginning of the vaccination campaign, or belonging to an age group over 65 years for which the vaccine dose being evaluated has been recommended, if different (it should be recommended for the entire age group). Birth year may be used instead of age in countries where vaccine recommendations are based on birth cohort, or where only year of birth is available.

● Permanent resident in the EU/EEA territory covered in the study (for each study site, according to the most recent information).

● Not residents of a nursing home/long term care facility (according to the most recent information at the beginning of the seasonal vaccination campaign).

● Received their first ever COVID-19 vaccine dose as part of an age-specific vaccination campaign (i.e., excluding those vaccinated before it was generally recommended in the corresponding age-group or, alternatively, excluding the first 5% of persons vaccinated within each age-group – for each 5-year age bracket- as these first vaccinees may not be representative of their corresponding age group).

● Completed primary vaccination at least 180 days before the start of the seasonal vaccination campaign.

● Has not received a COVID-19 vaccine dose, irrespective of the number of doses, in the last 90 days before the start of the seasonal vaccination campaign; has no documented SARS-CoV-2 infection (nor has been hospitalised due to COVID-19) in the 90 days before the start of the seasonal vaccination campaign (25), or; other criteria following the relevant national guidelines of each study site (for example, if seasonal vaccine is recommended irrespective of the time since last infection).

● Does not have inconsistent or missing data on vaccination (vaccination status unknown, any vaccination date is unknown, any vaccine brand is unknown, number of doses is unknown, interval between primary course first and second dose is shorter than 19 days, interval between complete primary vaccination and booster dose or between booster doses is shorter than 90 days, number of doses higher than recommended, received any vaccine brand not approved by EMA, or the combination of vaccine brands is not a recommended schedule -may vary by age group).

**Figures S1-4. Forest plots by age group and outcome, pooled estimates including the six study sites (Belgium, Denmark, Italy, Navarre-Spain, Portugal, and Sweden), from 1 June 2024 to 25 August 2024, VEBIS-EHR network.**

**Figure S1. Forest plot of autumn vaccination VE against hospitalisation related to COVID-19 among those aged 65-79 years.**

**
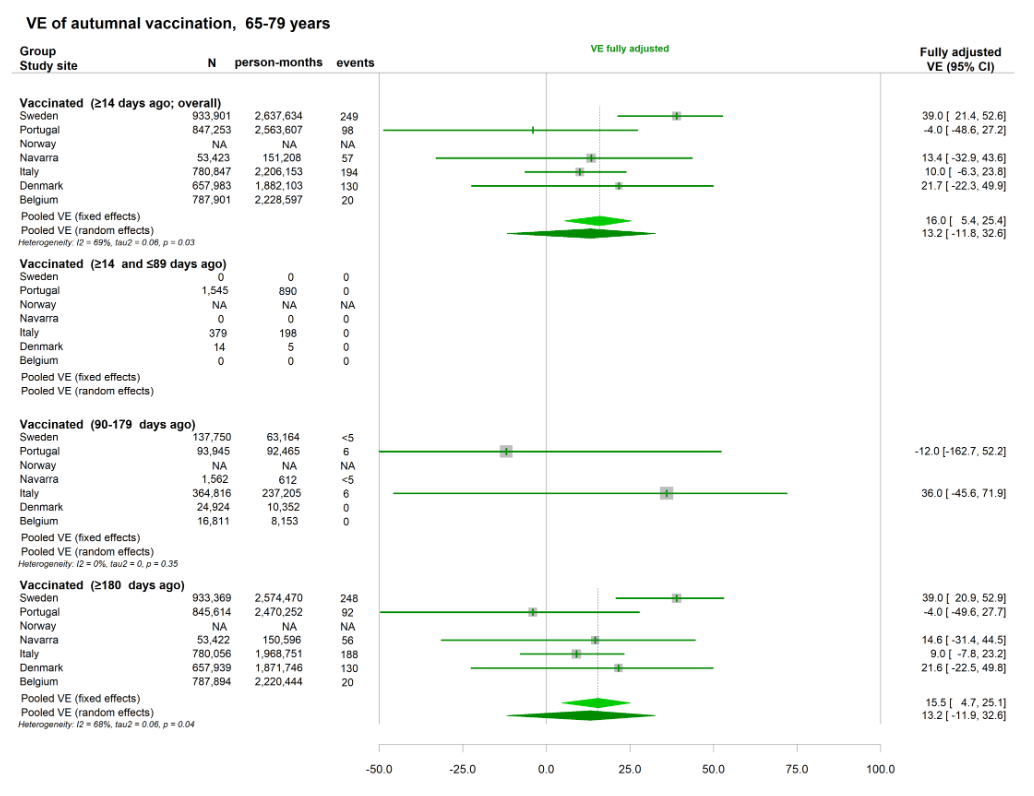
**

**Figure S2. Forest plot of autumn vaccination VE against hospitalisation related to COVID-19 among those aged 80 plus.**

**
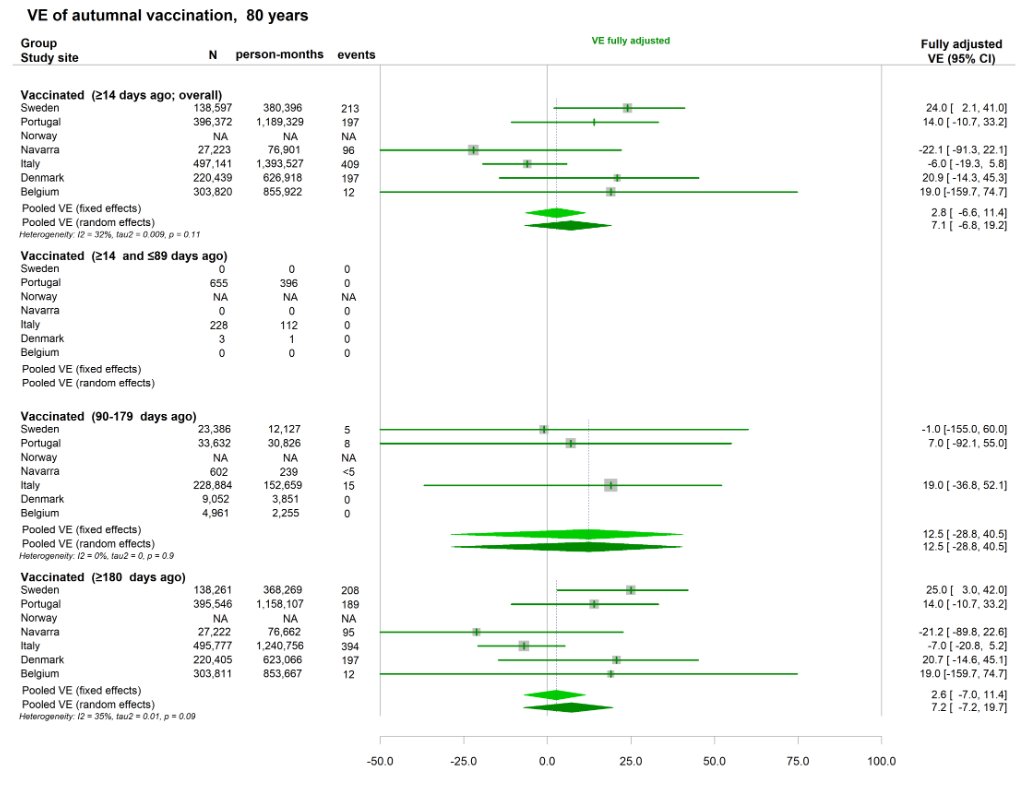
**

**Figure S3. Forest plot of autumn vaccination VE against death due to COVID-19 among those aged 65-79 years.**

**
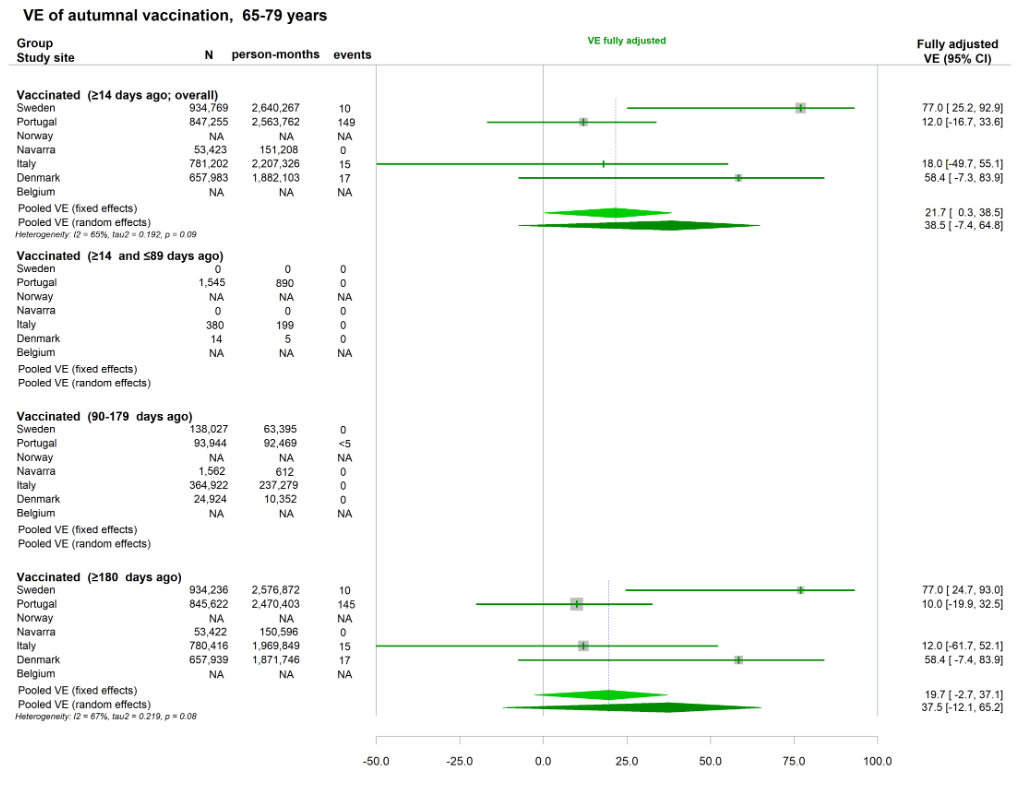
**

**Figure S4. Forest plot of autumn vaccination VE against death due to COVID-19 among those aged 80 plus.**

**
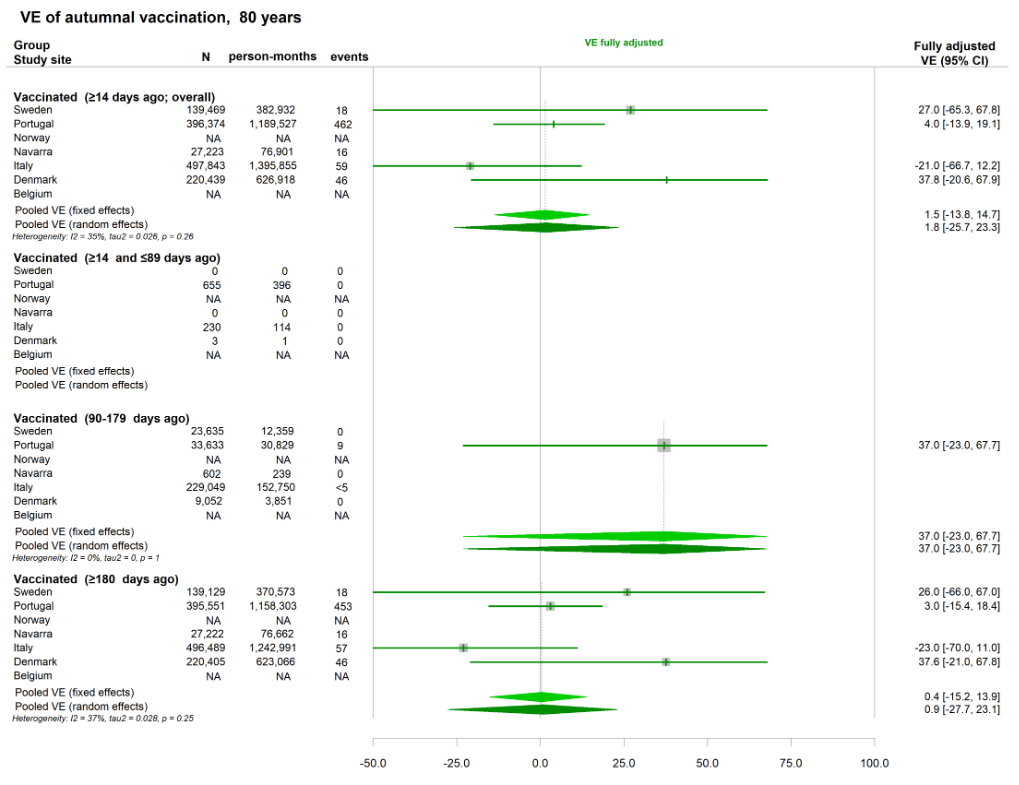
**
